# Factors Affecting the Compliance and Sway Properties of Tree Branches Used by the Sumatran Orangutan (*Pongo abelii*)

**DOI:** 10.1371/journal.pone.0067877

**Published:** 2013-07-02

**Authors:** Adam van Casteren, William I. Sellers, Susannah K. S. Thorpe, Sam Coward, Robin H. Crompton, A. Roland Ennos

**Affiliations:** 1 Faculty of Life Sciences, University of Manchester, Manchester, United Kingdom; 2 School of Biosciences, The University of Birmingham, Birmingham, United Kingdom; 3 Department of Musculoskeletal Biology II, Institute of Aging and Chronic Disease, University of Liverpool, Liverpool, United Kingdom; University of Kansas, United States of America

## Abstract

The tropical arboreal environment is a mechanically complex and varied habitat. Arboreal inhabitants must adapt to changes in the compliance and stability of supports when moving around trees. Because the orangutan is the largest habitual arboreal inhabitant, it is unusually susceptible to branch compliance and stability and therefore represents a unique animal model to help investigate how animals cope with the mechanical heterogeneity of the tropical canopy. The aim of this study was to investigate how changes in compliance and time of oscillation of branches are related to easily observable traits of arboreal supports. This should help predict how supports react mechanically to the weight and mass of a moving orangutan, and suggest how orangutans themselves predict branch properties. We measured the compliance and time of oscillation of branches from 11 tree species frequented by orangutans in the rainforest of Sumatra. Branches were pulled at several points along their length using a force balance at the end of a stiff rope, and the local diameter of the branch and the distance to its base and tip were measured. Compliance was negatively associated with both local diameter and length to the tip of the branch, and positively, if weakly, associated with length from the trunk. However, branch diameter not only predicted compliance best, but would also be easiest for an orangutan to observe. In contrast, oscillation times of branches were largely unaffected by local diameter, and only significantly increased at diameters below 2 cm. The results of this study validate previous field research, which related locomotory modes to local branch diameter, while suggesting how arboreal animals themselves sense their mechanical environment.

## Introduction

The locomotion of animals is often shaped by the environment they inhabit, and the physical properties of a habitat can drive locomotor adaptations [1]. The tropical arboreal environment is one of the most mechanically diverse habitats. It is a complex three-dimensional framework of morphologically variable supports whose mechanical properties vary [2]–[4]. This makes it a unique substrate and its arboreal inhabitants face locomotive challenges rarely shared by terrestrial animals. In particular the branches vary in two ways that will affect how animals locomote on them: compliance and oscillatory frequency.

Compliance (mN^–1^) can be defined best as the deflection (m) caused by a given force (N) and it is the reciprocal of stiffness. Within the tropical canopy, levels of compliance vary greatly. Large boughs close to the trunk of large emergent trees will have very low compliance, deflecting very little under the weight and movements of canopy dwellers. Compliance will increase towards the periphery of the tree crown; further away from the trunk, the diameter of the support decreases while the moment arm increases causing much larger perturbations to the branch [2], [4], [5]. Smaller, young and understory trees will have greater compliance because they have thinner more flexible branches and a more compliant trunk. Therefore it can generally be asserted that the highest degree of compliance will be at the tree periphery and inter-tree gaps. These canopy regions provide an interesting niche where some preferred food sources, like fruit, are readily available and energy whilst locomoting and foraging can be saved by gap crossing, instead of descending to and climbing from the forest floor to move terrestrially between trees [4].

The ground usually has essentially zero compliance, and running animals use the compliance of their own tendons to store energy and release it again during running [6], thereby improving the efficiency of their locomotion. Incorporating a small degree of compliance into a running track can, however, lead to enhanced performance in human runners and further reduce the cost of locomotion [7], [8]. In contrast, it has generally been found that branches have too much compliance to return energy to locomoting animals. The high compliance leads to low oscillatory frequencies, so when animals jump off branches they lose contact before the branch has time to recover [5], [9], [10]. The result is a reduced jumping performance and energy loss. Therefore, in general, it is assumed that higher compliance induces a locomotive cost to animals in the form of reduced performance and a higher energetic output [6], [10], [11]. In response, smaller primates face the problems of substrate variability and gap crossing in the canopy by adopting specialisations such as compliant gaits [12] and alternative leaping strategies [5], [13].

For larger animals such as the great apes the problems posed by varying compliance are more acute due to their greater body masses and therefore compliance poses a greater influence on their everyday lives. In order for large bodied animals to locomote safely and efficiently, they must be able to judge the stability and safety of supports with care as undue branch deformation or failure is much more likely to result in injury or even death [3], [4], [14]. The Sumatran orangutan (*Pongo abelii*) is the largest habitually arboreal great ape and it can be argued that it still lives in the ecological context of the ancestral great apes; making it an ideal study animal to understand the trials faced by both extant and extinct large bodied arboreal primates [2]–[4]. The Sumatran orangutan (*Pongo abelii*) has a body mass of around 40 kg for females and around 80 kg for males [15]. This makes them particularly susceptible to the changes in arboreal compliance. To deal with this their locomotion is characterised by slow movement, long contact times, and an impressively large array of locomotor postures [4], [16]. Orangutans have even been shown to utilise the compliance in vertical supports to lower the cost of locomotion by swaying trees back and forth [17] and they possess unique strategies of locomotion, moving slowly and using multiple supports to limit oscillations in compliant branches, particularly at their tips [4]. It has previously been suggested by Povinelli and Cant [3] that the larger body size and arboreal lifestyle of a great ape ancestor may have led to the evolution of an understanding of branch strength and compliance relative to their own weight, to facilitate planning and execution of arboreal locomotion. This may have been one of the evolutionary driving forces that led to some limited self-conception, where great apes or the common great ape ancestor develops a concept of their own body and the effect this has on mechanical environment around them. By studying the mechanics of the tropical canopy it may therefore be possible to develop a greater understanding of how orangutans perceive the changes in their arboreal surroundings and this could lead to further insights into the evolution of intelligence in humans and great apes.

Previous research suggests that orangutans may use branch diameter as a proxy for branch mechanical properties, both to judge compliance during locomotion [4], [18] and strength and rigidity for nest building [19]; they use differing locomotor modes on branches of different diameter, and fracture large and small diameter branches differently to make the structure and linings of their nests. Many researchers have also used branch diameter as a proxy for compliance when studying arboreal animals [2], [4], [11], [16], [18], [20]. Unfortunately there is little real evidence of a close correlation between diameter and compliance, mainly because of the complexity of the arboreal habitat and the practical difficulty in obtaining reliable biomechanical information from branches in the field. The position of an animal along a branch is also likely to affect the compliance it experiences. For this reason there are three alternative morphological traits of a point on a branch that an arboreal animal could use as a proxy for its compliance: branch diameter, distance from the tip of the branch, and distance from the base of the branch. The compliance of a branch will also depend on the material properties of the wood (i.e., the stiffness or Young’s modulus) of which it is composed. If little is known about branch compliance, even less is known about the times of oscillation of branches in the field, with or without arboreal animals. In a scaling study on trees, McMahon and Kronauer [21] showed that the natural frequency of oscillation of isolated branches of temperate species decreased with the square root of their length, as predicted by McMahon’s theory of elastic similarity. However, there are no field measurements for the branches of tropical trees or for trees loaded by the weight of an arboreal animal. Since the time of oscillation determines whether locomoting animals can obtain returns of elastic energy from an arboreal support, such measurements are also vital to fully characterise the mechanical environment of the arboreal niche.

The aim of this study therefore was to investigate how the compliance and time of oscillation of branches within the tropical rainforest canopy are related to four observable traits: diameter, distance from the tip of the branch, distance from the base of the branch, and Young’s modulus of the wood. We would expect compliance to be negatively correlated to all four traits. This research also allows us to test the assumption of previous authors that diameter is a good predictor of compliance. In addition we would expect the time of oscillation of a branch supporting an arboreal animal to be negatively correlated with local diameter. The results of this study will help future studies understand arboreal locomotion by providing direct measurements of the mechanics of the tropical canopy and since locomotion is a significant part of animals’ energy budgets, these results may help to develop a better understanding of their ecology. The research was accomplished by making direct measurements of compliance and diameter along branches of a variety of tree species frequented by orangutans in the rainforest of Sumatra. The research should expand our understanding of this complex environment and help us understand better how the locomotion of its arboreal inhabitants can be investigated in the field. Making and understanding actual measurements of compliance can also help us make more reliable simulations of the arboreal habitat in laboratory and zoo based investigations and help to better create the natural conditions for enrichment and reintroduction of captive animals.

## Methods

### Ethics Statement

Authorization to conduct research inside Indonesia was granted by Indonesian Ministry of Research and Technology (RISTEK). Permission to carry out research at Ketambe Reseach Station in the Gunung Leuser National Park was granted and approved through permits from The Directorate General of Forest Protection and Nature Conservation (PHKA), Badan Pengelola Kawasan Ekosistem Leuser (BPKEL), and Taman Nasional Gunung Leuser (TNGL).

### Tree and Branch Selection

The research was conducted over an 11-month period in the Gunung Leuser National Park in Aceh, Indonesia. The study area was a mixed tropical rainforest consisting of a large variety of tree species and abundant ground species. The forest shows stratification with the lower strata forming a closed canopy. The relative humidity is between 80 and 100% and the temperature fluctuates between 29–31°C [22]. Tree species were selected on the basis that they were populous in the environment and regularly used by orangutans in feeding or transport. In order to collect morphological and compliance measurements from the branches it was necessary to be able to access the canopy of the tree. This meant that only trees that could effectively and safely be accessed were used for the purposes of this study. Trees were climbed using the single rope technique and movement around the canopy was performed using the double rope technique [23], [24]. From each tree branches were then chosen for compliance testing and oscillation measurements. Table 1 gives the family and species, where known, for each tree tested. This is accompanied by the numbers of branches and individual points tested.

**Table 1:** Tree family, species and number of tests performed**.**

Family	Species	Branches	Points Tested	Young’s Modulus Tests
Clusiaceae	*Garcinia sp*	4	12	9
Cornaceae	*Mastixia trichotoma*	3	16	9
Dipterocarpaceae	*Parashorea lucida*	1	5	8
Moraceae	*Ficus benjamina*	2	9	16
Moraceae	*Ficus sp.*	3	11	9
Moraceae	*Ficus variegata*	2	7	6
Moraceae	*Ficus sumatrana*	5	21	7
Moraceae	*Ficus drupacea*	3	16	-
Elaeocarpaceae	*Elaeocarpus sp.*	1	3	7
Olacaeace	*Scorodocarpus borneensis*	3	9	9
Unknown Family	*Unknown species*	3	14	9

### Morphological and Compliance Measurements

Morphological measurements of branch lengths and branch circumference at given distances from the trunk, these varied from branch to branch to allow effective testing rope positioning. Lengths from the trunk were then measures directly using a measuring tape. Since all the branches were approximately circular in cross section, diameters were calculated by dividing circumference measurements by π. This gave a range of branch diameters between 0.01 m and 0.6 m.

Compliance (*C*) was calculated using equation 1 [10], where *dy* is the change in displacement and *dF* is the given force.
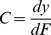
(1)


Compliance measurements were made at given points along a focal branch. At each point, a testing rope was passed over the branch and lowered to the ground. Once on the ground a series of knots a known distance apart were attached to the rope via a tensile steel ring. The first knot was then looped onto a mounted force gauge (Mecmesin, Advanced Force Gauge (AFG1000N)). The force gauge was anchored to the ground with the weight of a field assistant. The rope was then pulled down in increments by looping the knots sequentially onto the probe of the force gauge and the force required was measured. This gave a force/displacement curve. To prevent overestimation of compliance, we allowed for the compliance of the testing rope that was measured by a series of stretch experiments, allowing us to calculate rope compliance per unit length. The compliance of the length of rope that equalled the height of each branch was finally calculated and subtracted from the recorded compliance; this gave the true compliance for a given point along the branch [19].

### Oscillatory Frequency

The oscillatory frequency of the branch was determined by measuring the times of oscillations of the branch after it had been set swinging manually from the ground until oscillations were no longer visually detectable. We measured the time for a number of complete oscillations and divided by the number of oscillations to get the mean time for a branch.

### Young’s Modulus Measurements

To determine the stiffness of the wood material small branches were subjected to three point bending. Branch samples were collected from the canopy of test trees and returned to camp for testing. The samples were kept hydrated and tested within 1 week of extraction from canopy. Table 1 notes the number of tests for each tree species. The samples were all small peripheral sections of branch as the limitations of the portable three-point bending machine meant these samples had to be less than 3 cm in diameter and fell within a range from 0.6 cm to 2 cm. This limit is to reduce the effects of shear during testing, by ensuring a span-to-depth ratio of at least ≥20, since the maximum span of the testing equipment was 60 cm [25].

The three point bending apparatus was the same as that used in [19]. It consisted of a T shaped frame with a Mecmesin Advanced Force Gauge (AFG1000N) attached to the vertical mid bar via a screw attachment and stud bar which allowed the user to move the force gauge up and down the mid section of the frame. The samples were then placed on adjustable supports on the cross bar and the probe on the force gauge was hooked over the top of them. By turning the stud bar the force gauge was moved downwards and the sample was bent whilst the force was simultaneously measured. The displacement of the sample was recorded using a Mitutoyo Dial Indicator, which accurately measures small linear distances. From this a force displacement curve was generated, the slope of the initial linear region of this curve being the apparent stiffness (*(dF/dy)_app_*) of the branch.

In order to calculate the actual stiffness of the branch it is first necessary to remove the stiffness of the machine (*(dF/dy)_mach_*). This was calculated by performing a bending test on a steel rod of negligible compliance. From these two values it is possible to calculate the corrected stiffness (*(dF/dy)_cor_*) using equation 2 [26].
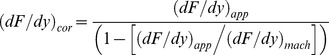
(2)


Once the corrected stiffness was calculated, the flexural rigidity (*EI*) of the branch was determined using equation 3 below, where *L* is the length between the supports [26], [27].
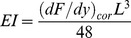
(3)


To calculate the flexural modulus of the wood of which the branch was composed, *EI* was divided by the second moment of area, *I*, [27] which for a cylindrical branch section is given by the formula.
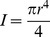
(4)


### Calculation of Branch Oscillation with Added Mass of Orangutans

A branch, which is supporting an orangutan or other arboreal animal, will oscillate more slowly than an empty branch because of the contribution of the animal’s mass to its effective inertia. To calculate the size of this effect, it is first necessary to calculate the effective mass *M_Branch_* at the point where the animal is supported [17]. This is the mass that would give the same oscillation time as the branch if it were acted on by a spring with the same compliance as that of the branch. It is given by the expression.
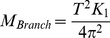
(5)


Where *K_1_* is the stiffness of the branch at that point, and *T* is the oscillatory time of the branch [17]. Once the effective mass is known it is possible to calculate the time of a complete branch oscillation (*T_Branch+Orang_*) with the added mass (*M_Orang_*), of an average-sized orangutan at a given point using the equation [17].
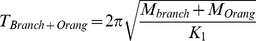
(6)


## Results

**Figure 1:**
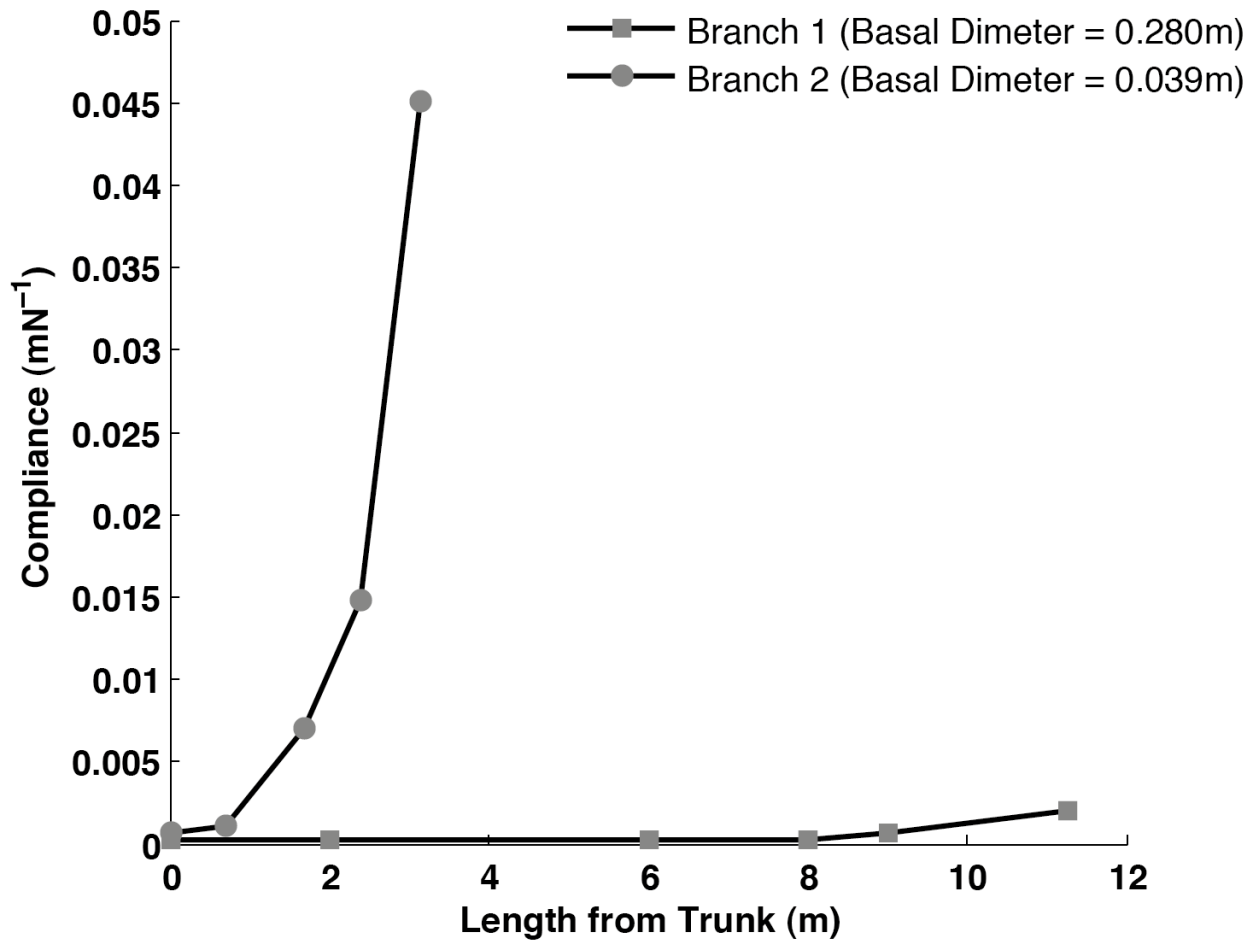
The compliance along two branches, one thick and long (branch 1) and one short and thin (branch 2).

Results of compliance were obtained at a total of 123 points along 30 branches from 11 different species. Along any one branch, compliance generally increased from base to tip, and longer, thicker branches were less compliant than thinner ones. Typical results from a long thick and short thin branch are shown in Fig. 1.

To determine which factor was the best predictor of compliance, we combined data from all the points and investigated how compliance was related to diameter, distance from the branch tip and distance from the trunk. We logged all data and carried out regression analyses, a procedure which is routinely carried out in mechanical scaling studies such as that of McMahon and Kronauer [21] and which is justified since morphological and mechanical properties are usually related to each other by power functions [28].

**Figure 2:**
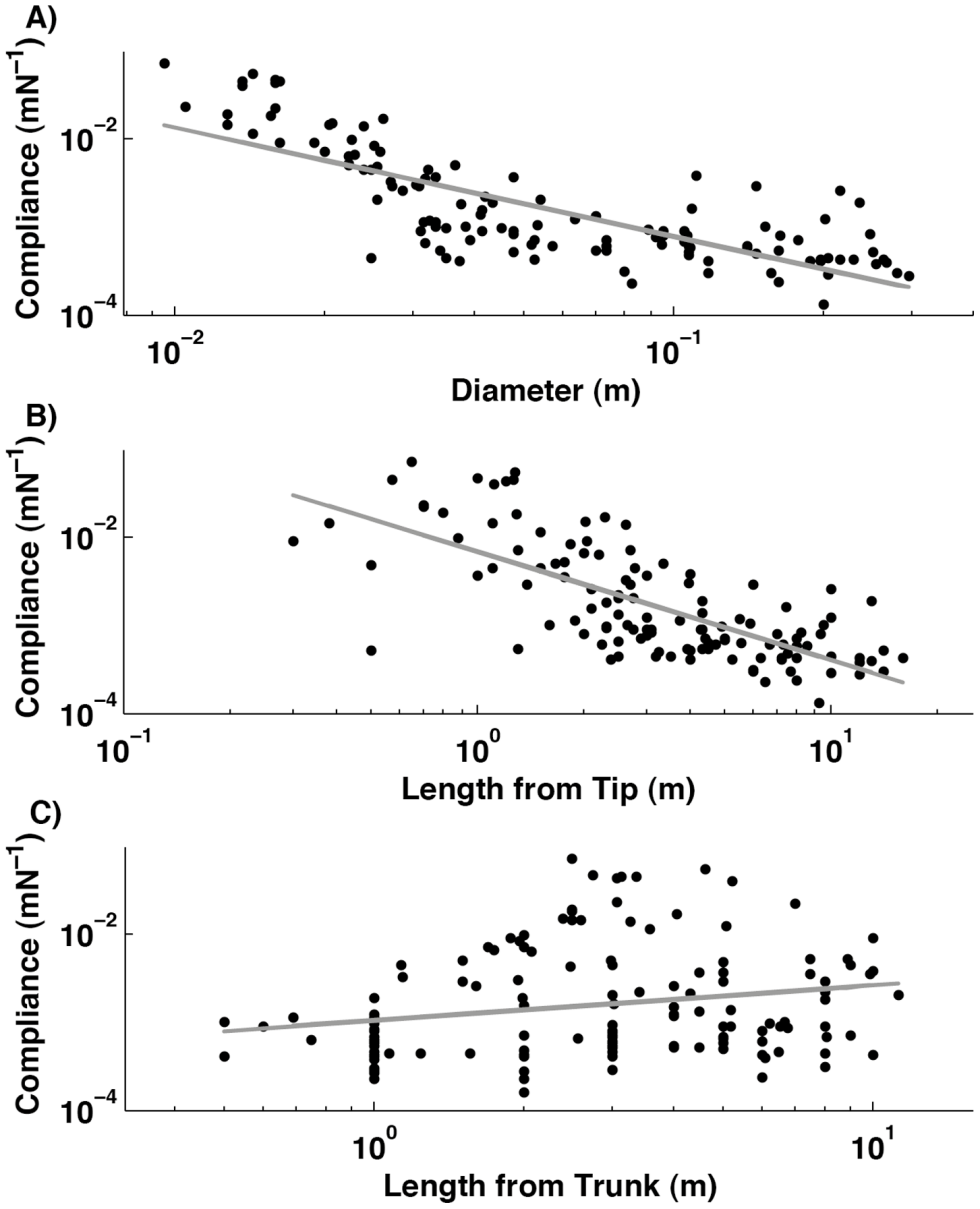
Log-log graphs showing how branch compliance is affected by different morphological properties. A) branch diameter (n = 123) B) length from branch tip (n = 123) and C) length from the trunk (n = 143).

Compliance (*C*) decreased as branch diameter (*D*) increased (Fig. 2A). Regression analysis of logged data demonstrated that this was a significant relationship, accounting for nearly 60% of the variability (t_121_ = –13.525, P<0.001, r^2^ = 0.599), the line of best fit being described by the equation.
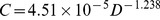
(7)


Compliance also decreased as length from the tip *L_bt_* increased (Fig. 2B). Regression analysis of logged data demonstrated that this was also a significant relationship (t_121_ = –11.468, P<0.001, r^2^ = 0.517), the line of best fit being described by the equation, though this accounted for around 52% of the variability.
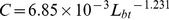
(8)


The relationship between compliance and length from the trunk (*L_tr_*) (Fig. 2C) was less pronounced and accounted for much less of the variation. Regression analysis of logged data showed a significant relationship (t_138_ = 2.547, P = 0.012, r^2^ = 0.038), the line of best fit being described by the equation, but this factor accounted for very little of the variability: less than 5%.
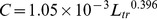
(9)


To determine whether a combination of the three factors could predict compliance even better, a stepwise multiple regression analysis was performed. This demonstrated that the two most reliable predictors of branch compliance were branch diameter (*D*), followed by the length from tip (*L_bt_*). The relationship can be best described by equation 10.
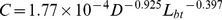
(10)


(F_2,120_ = 96.814, P<0.001, r^2^ = 0.617), though combining both diameter and length from the tip increased the explanatory power of the relationship by less than 2% compared with diameter alone.

**Figure 3:**
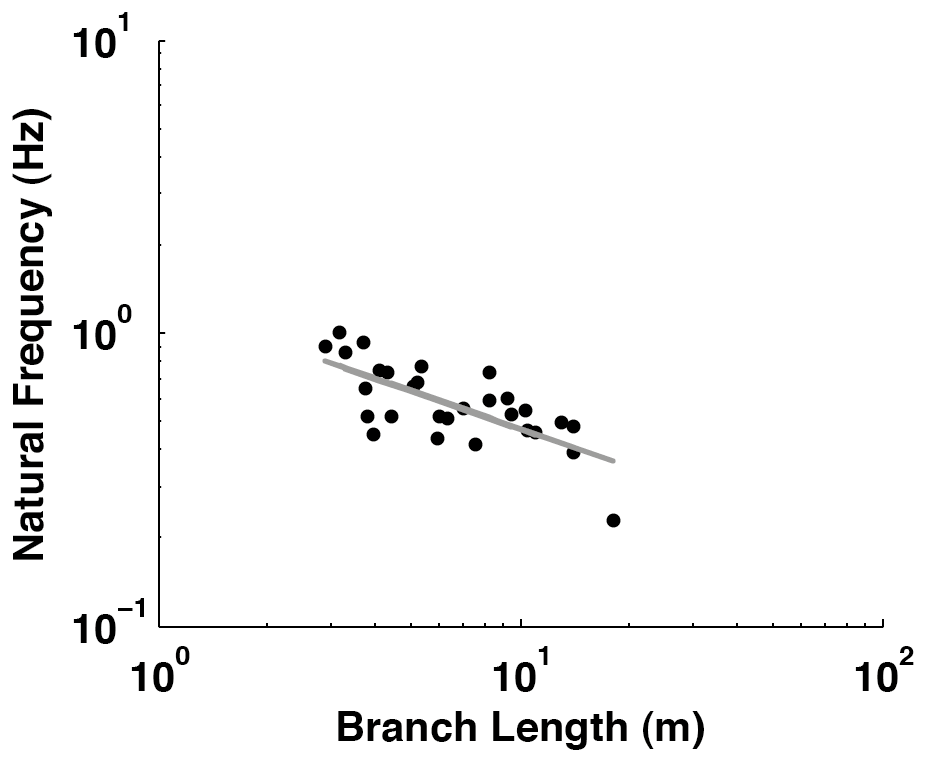
The relationship between natural frequency and branch length (n = 30).

Regression analysis of logged data demonstrated that there was also a significant negative relationship between the natural frequency (*f*) of branches and branch length (L) (Fig. 3) (t_28_ = –5.356, P<0.001, r^2^ = 0.515), the line of best fit being given by equation 11.
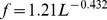
(11)


**Figure 4:**
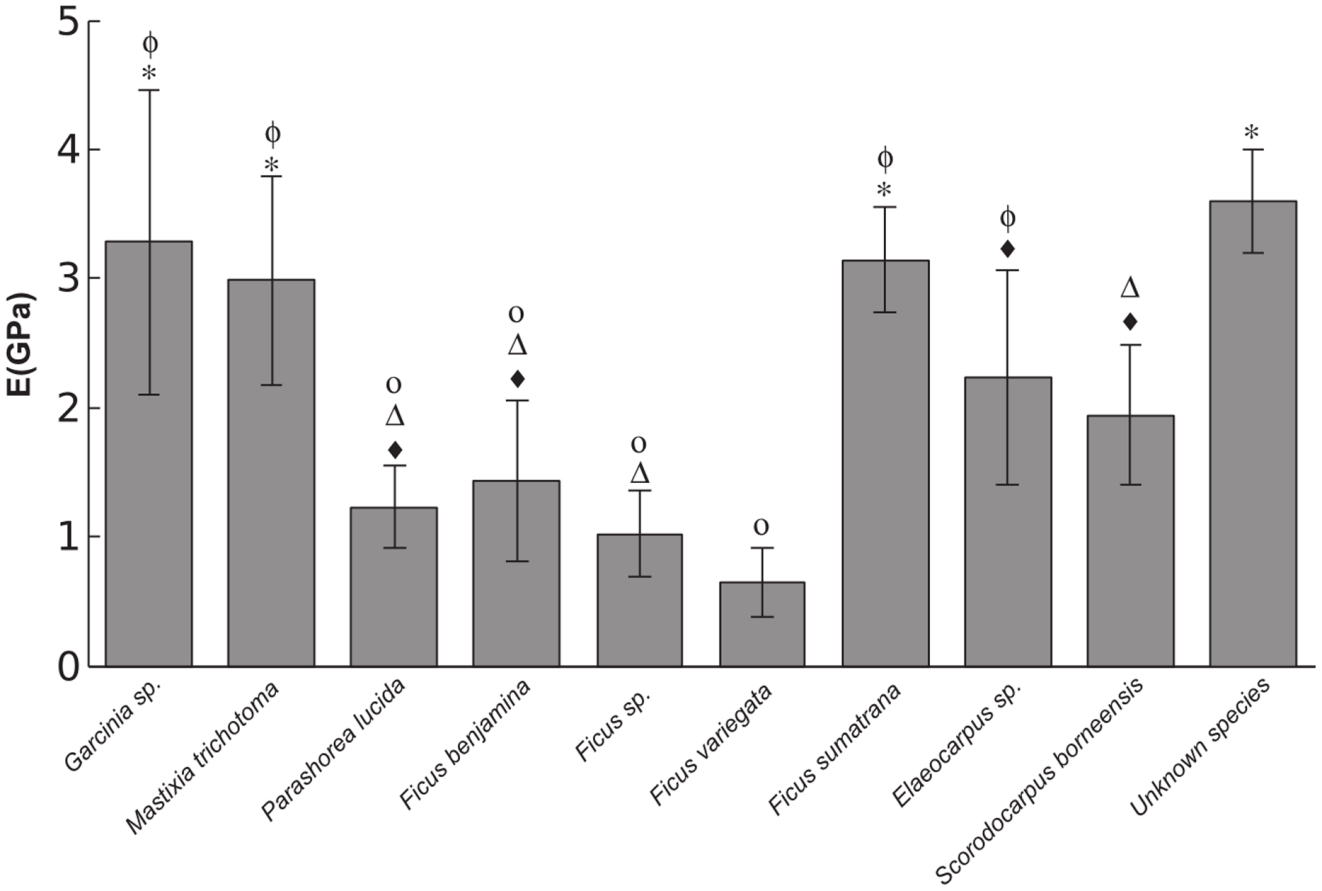
The mean Young’s modulus of 10 different tropical tree species frequented by orangutans. Error bars represent standard error. Species marked with the same marker are not significantly different from one another.

The results of the bending tests (Fig. 4) demonstrated that there were large differences in Young’s modulus of the wood of narrow branches of different tree species within the tropical canopy, though intraspecific variability was high. The results of a One-way ANOVA showed that these interspecific differences were significant (F_9,*78*_ = 22.684, P<0.001) and the results of a Tukey post hoc test are displayed in Fig. 4. Regression analysis of logged data revealed that there was a significant relationship between Young’s modulus and compliance (t_105_ = 2.764 P = <0.007 r^2^ = 0.059). However with such a low r^2^ value explaining less than 6% of the variation it would appear that Young’s modulus has very little influence on branch compliance.

**Figure 5:**
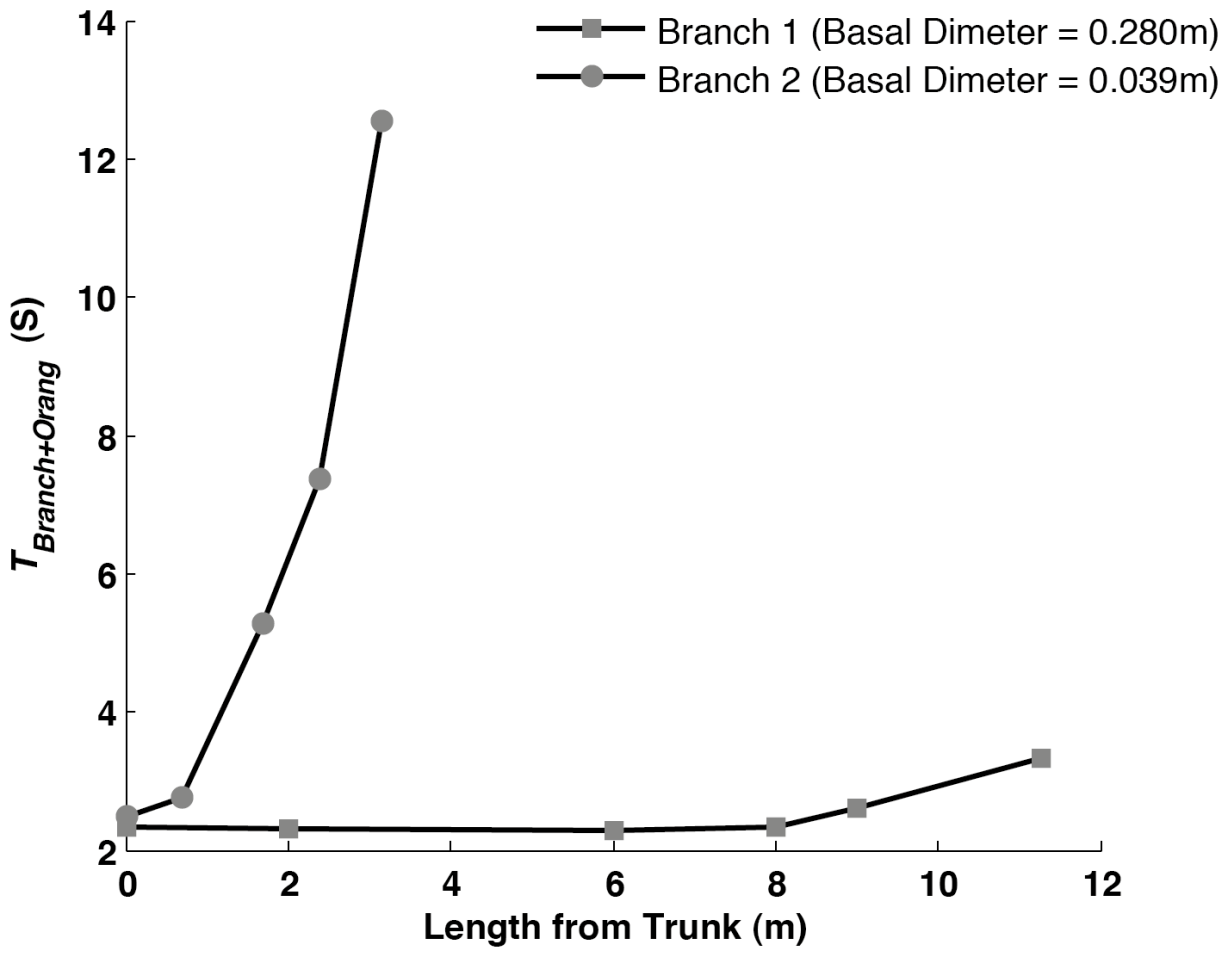
Demonstrating the time taken for a complete branch oscillation at different points along a branch towards the tip, with the added mass of a female orangutan (38 kg). This is done for two representational branches: one long and thick (branch 1) and one short and thin (branch2).

From the compliance measurements and the oscillatory frequency it is possible to calculate the time of oscillation for a point on a branch with the added mass of an orangutan (*T_Branch+Orang_*). Fig. 5 shows how *T_branch+Orang_* can change at different points, moving along the branch from the base towards the tip. This has been done for the two representational branches of Fig. 1: one thick and long (branch 1) the other small and thin (branch 2). In branch 1 the time of oscillation only rises at points approaching the branch tip, some distance from the trunk. However in branch 2, which is smaller and more compliant, the time of oscillation rises steeply much closer to the trunk due to the high compliance exhibited by the smaller branch.

**Figure 6:**
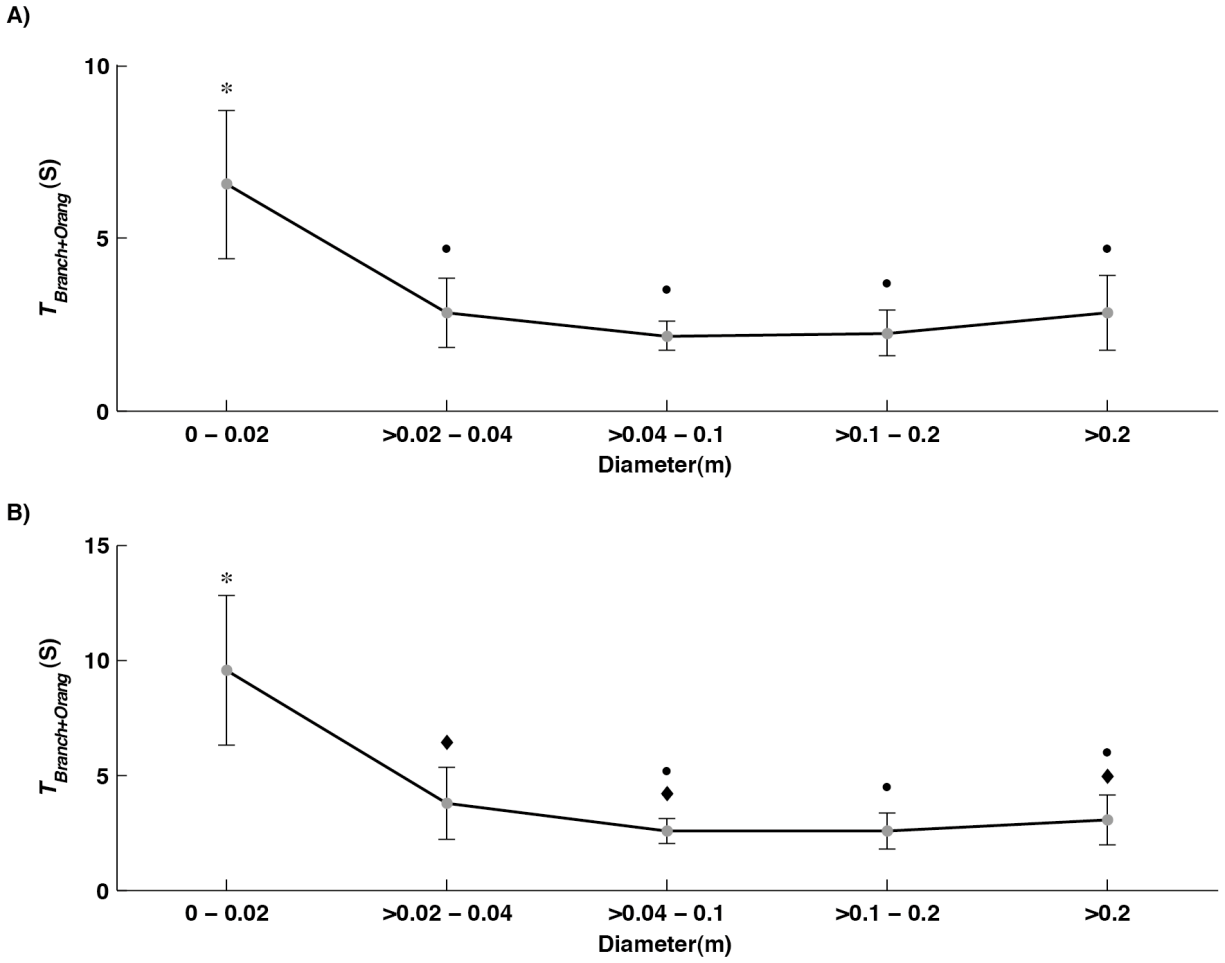
The mean time taken for a complete branch oscillation with the added mass of an A) female and B) male orangutan at different branch diameters. Error bars represent standard deviation. Groups marked with the same symbol show no significant difference.

Fig. 6 represents the calculation of average times of oscillation for particular ranges of branch diameter for an average female orangutan of 38 kg (Fig. 6A) and an average male orangutan of 86 kg (Fig. 6B). This demonstrates that the time for a complete oscillation rises as diameter decreases, but only for very narrow branches. As the data was found to be not normally distributed a Kruskal-Wallis test was performed on the data and this showed that for both females (χ^2^ = 49.4705 P<0.001) and males (χ^2^ = 53.2518 P<0.001) there was a significant difference between branches of different diameter. However, the results of a Nemenyi-Damico-Wolfe-Dunn post hoc test, presented in Fig. 6, show that only the thinnest branches, below 2 cm in diameter, have significantly longer times of oscillation.

## Discussion

The results of this study have demonstrated that though compliance is highly variable, with values ranging from 0.00013 to 0.07 mN^–1^, this variation can be predicted quite well from easily observable branch morphologies. This study also took *in vivo* measurements of the natural frequency of branch oscillation (Fig. 3) and related them to branch length. The results generated in the field were also used to see how the canopy substrate structures would react under the movement and added weight of the Sumatran orangutan, one of the largest canopy inhabitants (Fig. 5 and 6).

A major result of the study is that branch compliance has a significant relationship with all three morphological factors (Fig. 2): branch diameter, length from trunk and length from tip. However, there is a great difference between the predictive ability and potential usefulness of those factors. In this study, branch length from trunk was clearly the least effective at predicting levels of compliance and alone accounts for less than 5% of the variation (Fig. 2C). Length from the trunk is not very useful when trying to determine a single factor to predict compliance, as it does not relate well to other useful branch parameters, such as branch size or dimensions that have an effect on levels of compliance. An example of this is that small branches are compliant even close to their base (Fig. 1). Our results indicate that length from tip is a much more reliable tool for estimating the compliance of a branch, accounting for around 52% of the variation. Due to the way trees grow the branch tip is always the thinnest and furthest point from the trunk. Tree growth can be separated roughly into two main processes. Firstly the tree or branch lengthens through growth at the apical meristem and secondary growth occurs afterwards to widen and strengthen the branch by adding woody layers [29]. This generates the taper of branches and ensures the tip has the least amount of woody material at the greatest distance from the trunk, resulting in a higher degree of vertical displacement [30]. However, the tropical canopy is a complex meshwork of branches, lianas and epiphytes and it is unlikely, that a reasonable estimate of length from branch tip could be readily made as tree crowns are not easily identifiable as individual units [22]. This makes the identification of individual branch termini a sometimes-formidable task for researchers and arboreal inhabitants alike.

The single factor that allows for the most robust predictor of compliance is diameter. Diameter is a quick and easily identifiable trait of tree branches, its relationship with compliance was very significant as a single factor, and it accounted for the greatest degree of variation in the dataset at around 60%. Furthermore, adding distance from tip into a multiple regression analysis hardly improved the prediction of compliance; it increased the amount of variability predicted by less than 2%. Whilst it could be considered that only accounting for around 60% of the variation in compliance levels is somewhat low, this is probably close to the optimum predictive capability of a general rule for the tropical canopy. The wide variety in morphological traits, interactions between plants and differences between species means that a more accurate single predictive rule generated from field measurements would be hard to achieve. This supports the idea that arboreal animals may use diameter as a proxy for the mechanical properties of branches during locomotion, and can help justify the common practise of researchers of using diameter as a proxy for compliance [2], [4], [11], [16], [18], [20]. Diameter is likely to be a highly salient property of the branch for large arboreal inhabitants such as orangutans, as it can be readily and easily observed whilst locomoting or foraging in the canopy. The ability of orangutans to use diameter as an indicator of material properties of branches for the purposes of nest building has already been shown by [19].

The thin terminal branches subjected to mechanical tests (Fig. 4) showed marked interspecific variation in Young’s modulus. This difference is not unexpected as Young’s modulus is known to be quite variable between species [31] and can even change within individual trees as the wood ages [32]. When Young’s modulus was regressed against compliance we saw a significant relationship but this relationship had little to no predictive ability. This result indicates that knowledge of the Young’s modulus of the branch material is not paramount to understanding changes in canopy compliance. This adds more support to the argument that visually observable morphological traits such as diameter are a much more reliable predictor of branch compliance and could be readily utilised by large bodied arboreal inhabitants and researchers alike.

Like previous research on trees [21] our results show that branch natural frequency demonstrates a negative relationship with total branch length. McMahon and Kronauer [21] found the average frequency-length exponent of –0.59, which was steeper than –0.50 that would have been predicted by their elastic similarity model. Our observed exponent was lower at –0.43. However, the results of a t-test demonstrated this is not significantly different from the predicted exponent (t_26_ = 0.893, P = 0.005). The reasons for the slight differences in exponents between this study and that of McMahon and Kronauer [21] may be that unlike their “clamped branch” technique our results are from direct measurements taken from attached canopy branches which will be affected by the trunk compliance and the properties of the surrounding trees, branches, leaves or lianas.

Using the natural frequency of branches and the measured compliance at different points along a branch it is possible to model how an average branch may behave when supporting an average orangutan at a given diameter (Fig. 6). The time of oscillation of larger branch diameters actually stays relatively constant, being between 2 and 3 seconds, and it is only when the branch diameter falls below 2 cm that the time of oscillation increases significantly. This apparently counterintuitive result can be explained because we combined data from branches of a range of sizes. The time of oscillation will increase as an animal moves towards the tip of a branch, so *within* a branch time should be negatively correlated with diameter. However, longer, thicker branches will have longer times of oscillation than shorter thinner ones. Therefore *between* branches time of oscillation will be positively correlated with diameter. The two factors tend to cancel each other out and it is only at the tips of branches that the very low compliance of the narrow tips results in longer oscillation times. Noticeably there appears to be a much larger degree of variation on branches of smaller diameters as the deviations from the mean are larger when compared with the comparably stable larger regions generally found closer to the trunk. These findings are consistent with previous results that demonstrate that orangutans have developed unique locomotor strategies to deal with the high compliance that is common in branches below 4 cm in diameter, when oscillation times are more unpredictable and variable [4], [18]. Due to the dramatic and variable effects a large body size will have when locomoting or foraging in compliant regions of the canopy, orangutans cannot simply rely on stereotyped locomotion modes that do not take into account the structural nuances of the substrate like those of smaller canopy primates [3]. Instead, they have developed a range of non-stereotyped locomotor modes such as slow, unpatterned gaits with long contact times [4], [16], [33] and thus must cognitively solve the problems presented by large body size and variable arboreal compliance [3]. Of course, our results are unlikely to be fully representative of other forests. In particular, trees in temperate forests tend to have relatively thicker and hence less compliant limbs than those of the tropics, because of the higher winds in temperate areas [34], but they probably show a similar pattern of allometry. Studies investigating locomotion in different forests should therefore have to carry out some investigations of tree morphology at the local site.

Our results have indicated that the best observable trait for predicting branch compliance, both for arboreal animals and field researchers, is that of branch diameter. In contrast, oscillation times of branches are more-or-less unaffected by diameter, and only increase significantly at branch diameters below 2 cm. These results have therefore validated previous field studies and it is hoped will further stimulate both field and laboratory research attempting to understand how arboreal locomotion is affected by the complex mechanical environment of the arboreal niche.
